# Addressing diversity and inclusion challenges in global neuro-psychiatric and behavioral genomics research

**DOI:** 10.3389/fgene.2022.1021649

**Published:** 2022-12-13

**Authors:** Olivia P. Matshabane, Calandra G. Whitted, Laura M. Koehly

**Affiliations:** Social and Behavioral Research Branch, National Human Genome Research Institute, National Institutes of Health, Bethesda, MD, United States

**Keywords:** neuro-psychiatric genomics, behavioral genomics, equity, diversity, inclusion

## Abstract

Advancements in neuro-psychiatric and behavioral genomics offer significant opportunities for better understanding the human brain, behavior and associated disorders. Such advancements may help us prevent, manage and/or cure complex conditions. The serious challenge confronted by these disciplines however is diversity. Both fields lack diversity in terms of genomic reference datasets needed for discovery research, engagement of diverse communities in translational research and in terms of diverse and multidisciplinary scientific teams. This is a challenge because diversity is needed on all levels in order to increase representation and inclusion of all populations across the globe as we move research activities forward. The lack of diversity can translate to an inability to use scientific innovations from these fields for the benefit of all people everywhere and signifies a missed opportunity to address pervasive global health inequities. In this commentary we identify three persistent barriers to reaching diversity targets while focusing on discovery and translational science. Additionally, we propose four suggestions on how to advance efforts and rapidly move towards achieving diversity and inclusion in neuro-psychiatric and behavioral genomics. Without systematically addressing the diversity gap within these fields, the benefits of the science may not be relevant and accessible to all people.

## Introduction

Advancements in neuro-psychiatric and behavioral genomics are yielding innovations that have the potential to transform the lives of people living with neurodevelopmental, psychiatric and complex conditions. Despite many breakthrough developments, there is little progress in ensuring that participation in these fields is inclusive of people from all ancestral and geographical backgrounds (Nguyen et al., 2021). The NIH National Human Genome Research Institute’s strategic vision calls for genomic science to “strive for global diversity in all aspects of genomics research, commit to the systematic inclusion of ancestrally diverse and under-represented individuals in major genomic studies and to maximize the utility of genomics for all members of the public, including the ability to access genomics in healthcare” (Green et al., 2020). Currently, however, the fields comprising genomic science as a whole are not meeting these goals, with 86% of genomics studies being conducted on individuals of European descent (Fatumo et al., 2022) and even greater discrepancies in psychiatric genomics (Dalvie et al., 2015; Majara et al., 2021). There are several reasons for this across the discovery (T1) and translational research (T2—T3) pipelines (Woolf, 2008).

Specifically, in discovery research, genomic reference datasets do not have broad representation of participants inclusive of ancestral diversity leading to people from many cultural backgrounds and communities being under-represented in biomedical research. As well, such genomic reference datasets do not broadly include assessment of neuro-psychiatric phenotypes and environmental influences that may shape gene expression in the context of complex conditions with social and behavioral risk factors thereby limiting their utility for people globally.

Addressing the diversity gap is important for several reasons, which include 1) identifying previously unknown biological mechanisms, finding causal variants, assisting polygenic score probability assessment and understanding how environmental factors that affect neuro-psychiatric and behavioral disorders could inform treatments and interventions for these conditions (Reynolds et al., 2021) (T1); 2) successful implementation of emerging solutions in policy and practice, such as precision medicine (T2 and T3); and 3) for social justice reasons related to fairness and health equity (Bentley et al., 2017; Landry et al., 2018; Matshabane, 2021). With increased migration and environmental changes, addressing diversity challenges and fostering scientific methods which adapt to global needs ought to be at the forefront to successfully close the gaps in neuro-psychiatric and behavioral genomics. Here, we provide insights on the barriers to diversity in these fields and share four possible solutions to inform the way forward.

## Neuro-psychiatric and behavioral genomics

In this commentary, we distinguish between genetics—or the role that specific genes play in biology and disease—and genomics—or how gene-gene interactions and/or the interplay between genes and environment (i.e., environmental, social, and behavioral exposures) impact health and disease (Lea, 2009). Neuro-psychiatric genetic disorders are highly polygenic and reflect contributions from the interplay between environmental exposures and genetic variants; thus, many neuro-psychiatric disorders are genomic conditions. The field of neuro-psychiatric genomics aims to advance diagnostic approaches, as well as develop treatments and prevention strategies that are effective for addressing relevant disorders (Hoge & Appelbaum, 2012). Behavioral genetics includes neuro-psychiatric disorders with genetic or genomic etiology that result in behavioral phenotypes. As well, behavioral genomics may reflect the physical, social, environmental, and behavioral factors that can influence gene expression and how phenotypic differences may be observed especially among social groups like families that have shared genomic information (Samek et al., 2013). Below we discuss three barriers to diversity and inclusion in these disciplines.

## Barriers to diversity in neuro-psychiatric genomics and behavioral genomics

There are at least three key barriers to inclusive science in these fields. First, in relation to obtaining data—mistrust of scientists may be a dominant barrier for individuals of previously excluded communities participating in neuro-psychiatric and behavioral genomics research (Scharff et al., 2010; Erickson and Cho, 2011). People of color and other marginalized groups have historically experienced exploitation in the name of science (e.g., Havasupai Tribe case, Guatemala and Tuskagee syphilis studies) and these injustices have led to mistrust around decisions to participate in scientific research (Drabiak-Syed, 2010; Buseh, Stevens, Millon-Underwood, Townsend & Kelber, 2013). Second, people with neuro-psychiatric and behavioral conditions may have existing intersectional stigma and discrimination experiences which may influence their decisions to participate in genomics research (Lázaro-Muñoz et al., 2019). Participants may have concerns that participating could increase these experiences, which may be a relevant concern for those engaging in behaviors that increase their disease risk (e.g., those who eat in the absence of hunger and are overweight or those who have increased risk for psychiatric conditions and use substances like marijuana). Third, the lack of scientists who come from the same linguistic, cultural and genetic ancestral backgrounds as under-represented populations also contributes to research participants struggling to understand information communicated by scientists (Jooma et al., 2019).

## Four possible solutions

Scientific teams benefit from having social scientists who are trained in relational approaches and trust building to be at the forefront of neuro-psychiatric and behavioral genomics (Koehly et al., 2021). As social scientists working across these disciplines, we propose four possible solutions for enhancing diversity and inclusion in neuro-psychiatric and behavioral genomics: these include 1) awareness of environmental, social, and cultural factors relevant for different contexts, 2) enhancing genomic literacy, 3) creating and maintaining authentic partnerships with communities and 4) empowering communities through capacity building and deliberative engagements.

We ground these recommended solutions in reference to two multidisciplinary international collaborative studies situated at different stages in the discovery and translational pipelines: The Neuro-psychiatric Genetics in African Populations (NeuroGAP) network and the Families Sharing Health Assessment and Risk Evaluations (Families SHARE) project.

### Study 1: The neuro-psychiatric genetics in African populations network

NeuroGAP is a T1 or basic research effort which aims to increase representation of African populations in genome reference datasets, with a focus on neuro-psychiatric genetics studies (Stevenson et al., 2019). This goal is particularly important since it is known that modern humans originated in Africa (Trinkaus, 2005) and later migrated to different geographical locations, making Africa the continent with the greatest genetic diversity in the world. As such, African populations remain an important source of information for global genomics research. In contrast, African populations are represented in less than 2% of all current genomics datasets (Fatumo et al., 2022). The NeuroGAP network, which commenced in 2017 in four African countries (Ethiopia, Kenya, South Africa and Uganda) and is a collaboration with the Stanley Center for Psychiatric Research at the Broad Institute of MIT and Harvard University in the United States, is one example of a multidisciplinary international collaborative project aiming to increase representation of neuro-psychiatric genomic data of African people in global reference datasets (Wolde et al., 2021). This T1 initiative contributes to building equity through genomic discoveries that include under-represented African ancestral groups. The NeuroGAP network is split into the NeuroGAP-psychosis project which conducts research on the genetics of schizophrenia and bipolar disorder in South Africa, Ethiopia, Uganda and Kenya, and the NeuroDev study which conducts research in South Africa and Kenya, specifically focusing on genetics of childhood neurodevelopmental disorders (e.g., autism spectrum disorder, intellectual disability, attention deficit hyperactivity disorder and other cognitive and developmental delays) (de Menil et al., 2019). Given the lack of representation of people with different abilities in genomics and precision medicine research (Sabatello, Chen, Zhang & Appelbaum, 2019), the inclusion of people with neurodiversity through the NeuroGAP projects is a step towards representing views of people with a range of abilities. Across the NeuroGAP studies teams there are efforts to ensure that they maintain the following:

1. Awareness of environmental, social, and cultural factors. Through involvement of African participants from different parts of Africa, the NeuroGAP network is designed with an understanding of how different environmental, economic, social, linguistic and cultural factors stemming from the African context matter for neuro-psychiatric genomics research on the continent as well as how these factors may influence participants engagements in research. For example, the NeuroDev study involves parents with children who have neurodevelopmental conditions. Many of the parents do not have assistance to help with their special needs children while they participate in the research study. Local teams at the South Africa site therefore partnered with a hospital based non-profit organization that hosts experienced trainees who volunteer time to play with children and stimulate them with educational games while their parents are participating. This is an example of identifying what community members need and then addressing that need. Additionally, awareness of environmental, social, and cultural factors can assist in adapting research tools to best fit diverse African contexts. Another example, in the NeuroGAP studies, is that materials were carefully translated, adapted and validated for specific countries, with consideration of language, social values and norms, for ensuring the effectiveness of the science (Zieff et al., 2022). The NeuroDev study has therefore culturally adapted measurement scales for use among Kenyan children on the autism spectrum and the SNAP-IV instrument for ADHD assessment and validated the measure among South African children with neurodevelopmental disorders (Zieff et al., 2022). The NeuroGAP studies recruited and enrolled participants using the languages: Acholi, Afrikaans, Amharic, English, Kiswahili, Kigiryama, Luganda, Lugbara, Oromiffa/Oromigna, Runyankole, and isiXhosa (Atkinson, et al. 2022; de Menil et al., 2019).
2. Enhance genomic literacy. In both NeuroGAP studies, prior to obtaining consent, psychiatric research nurses and research assistants explain what genomics is, how genomics impacts one’s health, the specific goals that each project aims to achieve as well as risks and benefits of participating in the study. This is particularly important in contexts where individuals may not have genomic literacy. Following which, the psychiatric nurse or research assistant administers the University of California, San Diego Brief Assessment of Capacity to Consent Questionnaire (UBACC)—which is translated into their home language and adapted with one question related to participating in a genomic study—to test their understanding of the content and capacity to consent (see Campbell et al., 2017 with the English and isiXhosa version items of the questionnaire). Should they not pass this assessment, the above-mentioned information is explained again iteratively and after the fourth attempt of explaining and then completing the assessment, if they do not pass, they are not eligible and thus excluded from participating in the study. This assessment process ensures that the genomics and study information provided to participants during the consent process has been adequately understood—prior to participation in the study. Ensuring that participants (especially those with psychiatric conditions such as those enrolled in these studies) have some genomics literacy and do in fact understand the study information (including the potential risks and benefits) is important to ascertain ethical participation.
3. Create and maintain authentic partnerships with communities. During design stages of the NeuroGAP studies, participant priorities and recommendations based on past experiences were taken into consideration by the researchers, given that mostly the same researchers had engaged with the communities’ during preceding psychiatric genomics studies (i.e., see Gulsuner et al., 2020). These considerations involved respecting participants requests for limiting the length of the study battery and minimizing intrusive sample collection (e.g., through collecting saliva instead of blood). Because researchers in the project had worked with the communities, including through previous formation of a population-specific community advisory board (CAB) for psychiatric genomics research in South Africa (Campbell et al., 2015) and through a mental health literacy day with South African Xhosa people in the Eastern Cape Province (Campbell et al., 2021), they could draw on those experiences to ensure the development of procedures and methods that consider contextual and cultural nuances. The processes followed in these studies are important examples for other researchers to consider when conducting neuro-psychiatric genomics research with under-represented and under-served communities. Notably, the development of a CAB involving the researchers and selected community members, can be one method to promote authentic partnerships and engagement with communities. In this example, the CAB assisted researchers with input in relation to consent processes, tool development, recruitment strategies and informed them on methods to minimize potential harm/stigma and discrimination towards community members (Stevenson et al., 2019). Additionally, results of the study will be shared with local CABs which is a demonstration of further seeing the communities as partners while honoring and respecting the dignity of communities.
4. Empower communities through capacity building and deliberative engagements. Respecting communities through the ways in which research is conducted is particularly important. One way of doing that is by ensuring people from those communities are empowered to actively participate in scientific engagements being conducted in their context. At the level of diversifying scientists in collaborative international genomics studies the NeuroGAP Global Initiative for Neuro-psychiatric Genetics Education and Research (GINGER) Program trains and mentors early-career investigators from the specific African countries where the projects are being conducted (i.e., Uganda, Kenya, South Africa and Ethiopia) to ensure that they develop the capacity to actively provide intellectual leadership of neuro-psychiatric genomic studies in Africa (van der Merwe et al., 2018; Martin et al., 2022). The GINGER program will ultimately contribute to promoting diversity and inclusion of African scientists in global neuro-genomics collaborations. Doing so can contribute to building trust and ensuring that participants better understand neuro-psychiatric genomics (e.g., through having people who speak the same language or come from the same cultural background and/or community as them—engaging with them about being involved in neuro-psychiatric genomics research).

### Study 2: Families sharing health assessment and risk evaluation

With respect to T2 and T3, or translational research, inclusion of minoritized populations in behavioral genomics research requires critical considerations for design and implementation strategies. Families SHARE is a toolkit, comprised of a workbook, community education program, and video, with the goal of increasing families’ genomic health literacy as it relates to family health history of complex conditions with behavioral risk factors and encourage family engagement around conversations about risk and risk-reducing behaviors (Koehly et al., 2015; de la Haye et al., 2021; Wilson, et al., 2016). The Families SHARE program has been adapted to multiple geographical and cultural contexts and is an example of a scientifically and culturally diverse collaboration which has allowed translation to reach various populations including White and historically under-represented populations in biomedical research in the United States, immigrant and ethnically diverse communities in Australia, and communities in rural Nigeria, Africa. Moreover, Families SHARE is being used as an educational module for a home visitation healthcare program in southeastern Florida, demonstrating successful dissemination of the toolkit. To improve community utility and engagement with the toolkit, as researchers leading this project we entered each community with cultural humility with the goal of tailoring toolkit components to specific community needs. This involved establishing research partnerships with team members who brought expertise in a variety of domains, including site specific expertise and social capital, and employed the following strategies:

1. Awareness of environmental, social, and cultural factors. First, we had to assess and understand for each geographical location various environmental, social, and cultural factors that would impact the utility and acceptability of the Families SHARE toolkit. For optimal results, understanding these factors required a collaborative team with diverse expertise to provide input on the design of processes, procedures, and materials (e.g., language differences and gender roles). Research teams included, for example, members with content, language, and site-specific expertise, community partners, and design specialists, to maximize impact in the community setting. As well, listening sessions with and direct feedback from community members was invaluable. For example, working in rural communities in Nigeria required an understanding of important cultural differences that needed to be integrated into the toolkit, resulting in a language appropriate version (Hausa language), recognizing religious cultural representation in the Families SHARE materials (e.g., hijab worn by women), and depicting and recommending regionally and culturally appropriate examples of fruits and vegetables or physical activity (e.g., mango, cabbage, bicycling) to properly respect the norms of the community.
2. Enhance genomic literacy. The Families SHARE toolkit focuses on five complex diseases (i.e., heart disease, type 2 diabetes, colon cancer, breast cancer, and prostate cancer) and includes disease fact sheets that define the condition along with associated risk factors and health guidelines for risk reduction and early detection. Given the focus on genomic literacy, a family health history-based risk algorithm is provided that demonstrates how disease patterns within families are associated with increased disease risk. Participants are provided with a personalized three generation pedigree which they use as they walk through the family history-based risk algorithm for each disease. Doing so, introduces the information in a personalized way. Currently, the toolkit includes a workbook written at a grade 8 reading level, a moderators’ guide for a community education program, a short instructional video, and a curriculum guide for health educators. While the Families SHARE toolkit was developed to enhance genomic literacy through its use, the needs of each community setting were considered, and components of the toolkit were adjusted to address those needs. For example, screening and behavioral recommendations were adjusted to be consistent with guidelines that vary by country and public health information sources provided were specific to the country or region where the toolkit was being used. The workbook has been translated into four languages—Hausa for use in rural Nigeria—and English, Spanish, and Haitian-Creole to serve communities within the United States. Through listening sessions with key stakeholders and community members, we identified a need for training materials grounded in a train-the-trainer framework such that the toolkit can be used by community health workers and clinicians, patient navigators, teachers, and family genomics educators.
3. Create and maintain authentic partnerships with communities. Authentic community partnerships are created through engagement of community members as research partners, leveraging unique expertise of community stakeholders. The Florida site of Families SHARE showed success through engagement of existing community health coalitions and churches to improve access to engage participants in the study. Efforts in the Washington DC area include a partnership with a local academic institution embedded within the community and research navigators who know community residents. We developed partnerships with local community organizations. The community-based and family-based educational components of Families SHARE, which were tailored to the specific needs of the communities by the study team embedded within, served to empower participants to voice their opinions and ideas for how the toolkit could be used within their own family, given the unique social roles that each member takes on.
4. Empower communities through deliberative engagements. Utilizing a network of community partners is important for establishing a two-way system of trust between participants and researchers. Throughout the Families SHARE dissemination process, factors such as community context and location have been considered. The partnerships can enable future research—such as increase participation in sequencing research, returning results, and other educational and intervention opportunities—as they represent trusted pathways for genetic and genomic-related activities. Moreover, participants who engaged in Families SHARE at the Washington, DC study site reported feeling empowered by using and sharing the Families SHARE workbook with family and healthcare providers (de la Haye et al., 2021). This points to the importance of bi-directional relationships such that scientific teams are supporting communities and communities are benefiting from the research.

### Improving community diversity

**FIGURE 1:**
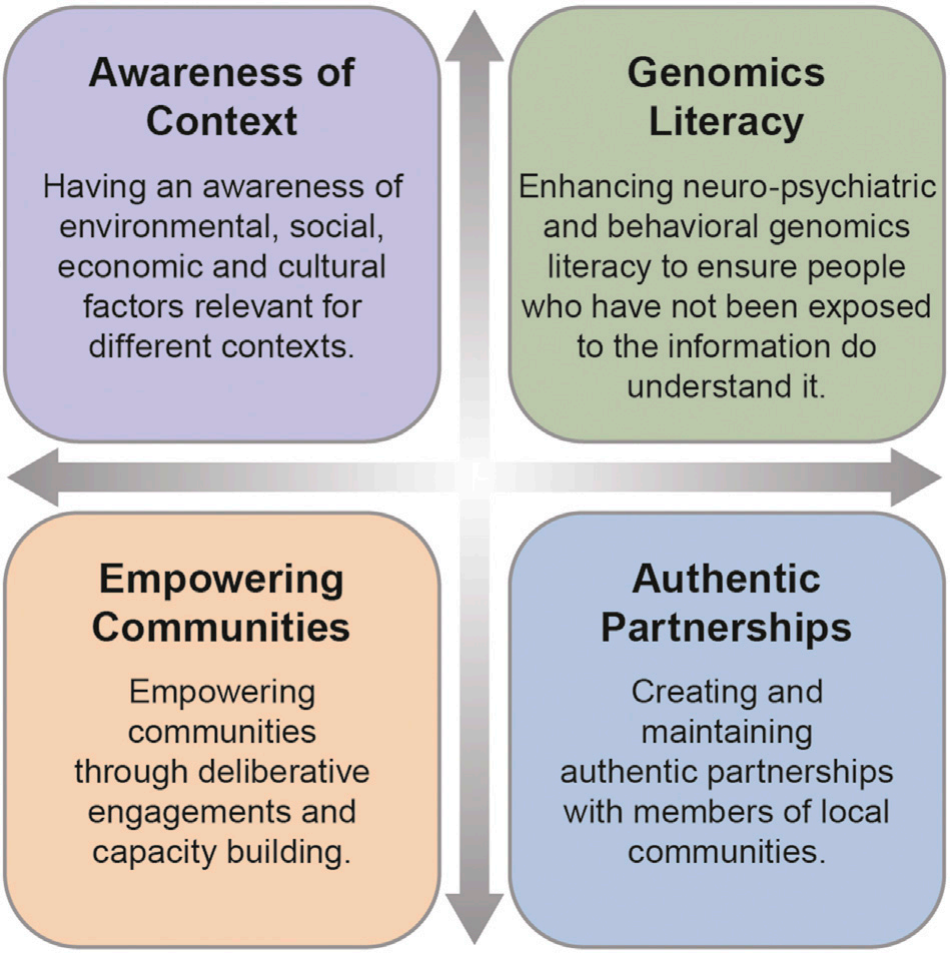
Four solutions for addressing diversity and includion in neuro-psychiatric and behavioral genomics.

Both these studies have demonstrated that empowering communities through equipping them to confidently engage and negotiate with researchers is an important step towards creating more equitable partnerships and leveling power dynamics in scientific research (Lemke, et al., 2022). We have presented four solutions on how to do this in the genomic patient population and the workforce (see Figure 1). In addition, we believe transparency, clear communication and education about short and long-term goals of research with an opportunity for deliberation, sustainability planning, capacity building and dissemination represent best practices for addressing diversity and inclusion challenges in neuro-psychiatric and behavioral genomics studies. This diversity needs to not only be defined in terms of race and ethnicity, but also needs to include other under-represented groups such as people with disabilities and members of the LGBQIA+ community, whose voices are often ignored. One way of implementing these best practices is through a Community Based Participatory Research (CBPR) approach which, if employed successfully, creates and maintains true partnerships between communities and researchers such that each is engaged in decisions regarding research design that meet community needs (Skinner et al., 2015). As well, each of these examples reflect the strength of a diverse research team, with team members from a breadth of disciplines and experiences, including individuals from the communities within which we work, each of whom bring an important perspective to addressing the diversity and inclusion gaps within the field. More exemplars such as these are crucial for the success of the current agenda.

### Improving workforce diversity

**FIGURE 2:**
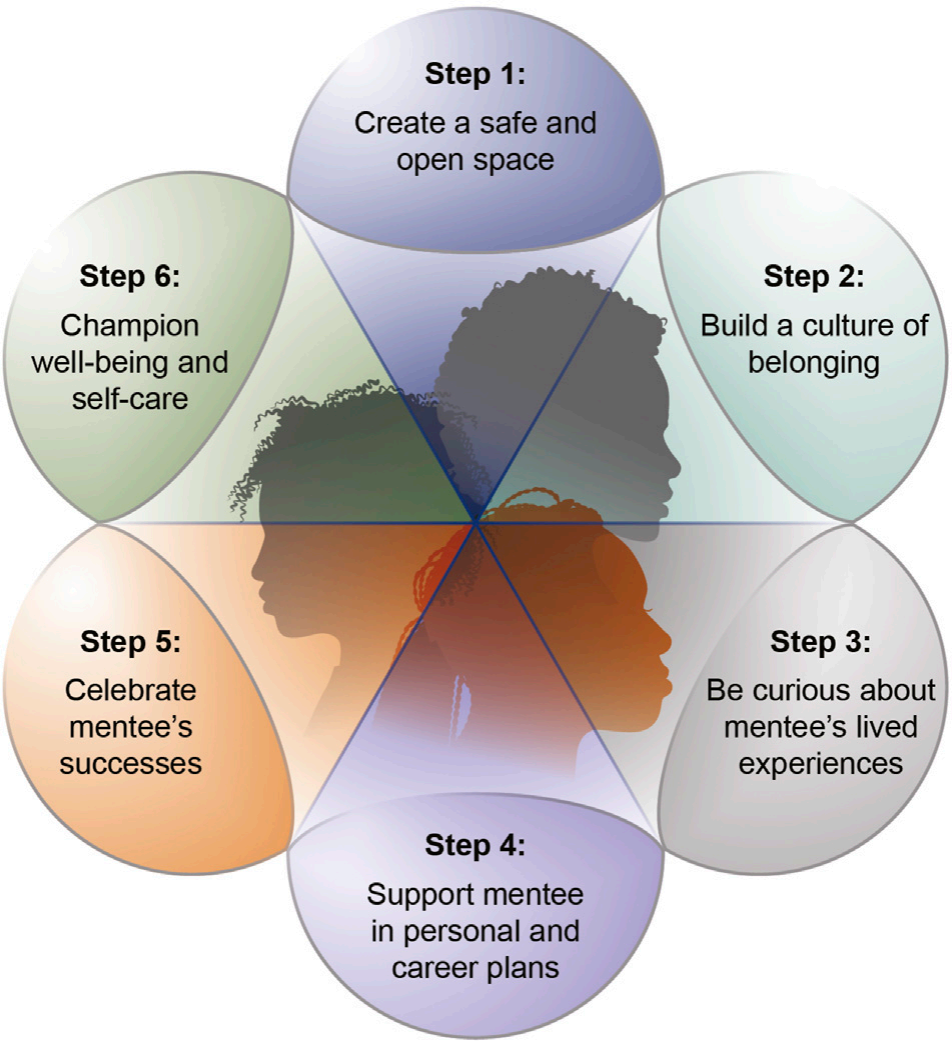
Six steps of intentional mentoring to enhance diversity and inclusion in neuro-psychiatric and behavioral genomics.

Importantly, our proposed solutions to increasing diversity and inclusion of ancestrally diverse and under-represented populations in neuro-psychiatric and behavioral genomics studies rely upon a diverse scientific workforce. Scientific workforce diversity (including in relation to different disciplines) has important implications in reaching study participants from diverse backgrounds as well as enhancing innovation in scientific endeavors given the unique personal experiences and varied disciplinary lenses needed to address both discovery and translational questions (Valantine and Collins, 2015). Unfortunately, given the lack of diversity in STEMM (Science, Technology, Engineering, Mathematics, and Medicine) fields, it is difficult for emerging scientists to identify mentors who share a common background as them (Haddad et al., 2021). Again, this is event for example in the case for people with disabilities in higher education and STEMM (see Moon, et al., 2012). A scoping review on the inclusion of people with disabilities in higher education revealed that this group is largely excluded, even within the ways policies for equity, diversity and inclusion are operationalized in many institutions (Wolbring & Lillywhite, 2021). Similar trends are reported on people from the LGBTQIA + community, who are also often excluded (Cech & Waidzunas, 2021). Resultantly, students and trainees often don’t have the opportunity to identify senior members of academic and non-academic staff who can be role models or effective mentors. Moreover, people who have a disability or are part of the LGBTQIA+ community often fall into other minoritized groups and therefore experience even more difficulty with identifying role models and mentors because of their intersectional lived experiences. To address this challenge, it is important to promote a training environment grounded in intentional mentoring which identifies and includes mentors from diverse backgrounds. Intentional mentoring is personalized, with mentors tailoring their mentoring approach to mentees’ needs to ensure that emerging scientists from under-represented groups thrive (Nadal, Whitman, Davis, Erazo & Davidoff, 2016; Shuler et al., 2021; Singh, Bhambhani, Skinta & Torres-Harding, 2021; Murray et al., 2022). As outlined in Figure 2, six steps to intentional mentoring include: 1) creating a safe and open space, 2) building a culture of belonging, 3) being curious about mentees’ lived experiences, 4) supporting mentees in personal and career plans, 5) celebrating mentees’ successes and 6) championing wellbeing and self-care.

## Conclusion

Taken together, environmental, social, and cultural factors are important for how genomic information is viewed, accessed, and used by individuals in various parts of the world. People in low-resourced contexts, including those who have had no or limited access to education, healthcare and basic needs, may not be able to access or use genomic information in ways that can better their lives (i.e., in healthcare) as those in more affluent contexts could if we don’t successfully address the challenge of diversity. Therefore, we recommend that researchers identify these underserved communities, seek out key local stakeholders in communities, engage with stakeholders and identify what barriers need to be addressed together with the community to improve representation. Doing so can ultimately level the power dynamics between researchers and community members, which could create an opportunity for authentic partnerships that are grounded on cultural competence and humility (Matshabane, Mgweba-Bewana, Atuire, de Vries & Koehly, 2022). This how we can inform best practices which will benefit all people globally.

### Resources from studies discussed in this paper

1. NeuroGAP Psychosis: https://www.broadinstitute.org/stanley-center-psychiatric-research/neurogap/neurogap-psychosis
2. NeuroDev: https://www.neurodevproject.org/
3. GINGER: https://www.broadinstitute.org/stanley-center-psychiatric-research/neurogap/global-initiative-neuropsychiatric-genetics-education-research-ginger
4. Families SHARE: https://www.genome.gov/research-at-nhgri/Projects/Families-SHARE#:∼:text=risk%20for%20diseases.-,Overview,risk%20for%20many%20different%20diseases.

### Other relevant resources

1. Guidance for genetics and genomics researchers on adopting community-engaged research approaches throughout the research lifecycle: https://www.cell.com/action/showFullTableHTML?isHtml=true&tableId=tbl1&pii=S0002-9297%2822%2900357-3
2. ASHG Diversity and Inclusion Task Force Action Plan: https://www.ashg.org/about/committees/diversity-inclusion-task-force/ditc-action-plan/.

## Data Availability

The original contributions presented in the study are included in the article, no additional data is available, further inquiries can be directed to the corresponding authors.
